# EZH2 protein expression in normal breast epithelium and risk of breast cancer: results from the Nurses’ Health Studies

**DOI:** 10.1186/s13058-017-0817-6

**Published:** 2017-03-02

**Authors:** Francisco Beca, Kevin Kensler, Benjamin Glass, Stuart J. Schnitt, Rulla M. Tamimi, Andrew H. Beck

**Affiliations:** 1 Cancer Research Institute, Beth Israel Deaconess Cancer Center, Boston, 02215 MA USA; 2 Department of Pathology, Beth Israel Deaconess Medical Center, 330 Brookline Ave, Boston, 02215 MA USA; 3 Harvard Medical School, Boston, 02215 MA USA; 4 Department of Epidemiology, Harvard T.H. Chan School of Public Health, Boston, MA 02115 USA; 5 Channing Division of Network Medicine, Department of Medicine, Brigham and Women’s Hospital and Harvard Medical School, Boston, MA 02115 USA

**Keywords:** Benign breast disease, Breast Cancer, Risk, EZH2, Nurses’ Health Studies

## Abstract

**Background:**

Enhancer of zeste homolog 2 (EZH2) is a polycomb-group protein that is involved in stem cell renewal and carcinogenesis. In breast cancer, increased EZH2 expression is associated with aggressiveness and has been suggested to identify normal breast epithelium at increased risk of breast cancer development. However, the association between EZH2 expression in benign breast tissue and breast cancer risk has not previously been evaluated in a large prospective cohort.

**Methods:**

We examined the association between EZH2 protein expression and subsequent breast cancer risk using logistic regression in a nested case-control study of benign breast disease (BBD) and breast cancer within the Nurses’ Health Studies. EZH2 immunohistochemical expression in normal breast epithelium and stroma was evaluated by computational image analysis and its association with breast cancer risk was analyzed after adjusting for matching factors between cases and controls, the concomitant BBD diagnosis, and the Ki67 proliferation index.

**Results:**

Women with a breast biopsy in which more than 20% of normal epithelial cells expressed EZH2 had a significantly increased risk of developing breast cancer (odds ratio (OR) 2.95, 95% confidence interval (CI) 1.11–7.84) compared to women with less than 10% EZH2 epithelial expression. The risk of developing breast cancer increased for each 5% increase in EZH2 expression (OR 1.22, 95% CI 1.02–1.46, *p* value 0.026). Additionally, women with high EZH2 expression and low estrogen receptor (ER) expression had a 4-fold higher risk of breast cancer compared to women with low EZH2 and low ER expression (OR 4.02, 95% CI 1.29–12.59).

**Conclusions:**

These results provide further evidence that EZH2 expression in the normal breast epithelium is independently associated with breast cancer risk and might be used to assist in risk stratification for women with benign breast biopsies.

**Electronic supplementary material:**

The online version of this article (doi:10.1186/s13058-017-0817-6) contains supplementary material, which is available to authorized users.

## Background

Prior studies have elucidated a variety of clinical, epidemiologic, radiologic and histopathologic features associated with an increased risk of breast cancer. However, our understanding of breast cancer risk factors and carcinogenesis in the normal breast is still rudimentary, limiting our ability to predict breast cancer risk accurately. Currently, germline mutations in high penetrance cancer predisposition genes (i.e. *BRCA1*/*2*), family history, and mammographic density have major ability to predict breast cancer, with other factors such as reproductive history playing a smaller role in determining risk [1–3]. Among the breast tissue-based predictors of future cancer risk, atypical hyperplasia (atypical ductal hyperplasia and atypical lobular hyperplasia) is the strongest predictor and is associated with a three to fivefold increase in the risk of breast cancer [4, 5]. Additionally, some tissue-specific biomarkers have been associated with breast cancer risk in specific subsets of women, such as insulin-like growth factor 1 receptor (IGF1R), and more recently, the combination of Ki67 and estrogen receptor (ER) expression in a subset of women [6–8]. However, additional biomarkers to stratify breast cancer risk are needed.

Enhancer of zeste homolog 2 (EZH2) is a core protein of the polycomb-repressive complex 2 (PRC2) that controls dimethylation and trimethylation of Lys27 of histone H3 (H3K27me3), a marker linked to transcriptional silencing. Due to its role in regulating fundamental cellular processes, such as cell cycle regulation, senescence and differentiation, EZH2 has been thought to contribute to malignant transformation. However, this view has been challenged and it has been suggested that high EZH2 is a consequence rather than a cause of cancer [9].

More relevant to this study, the expression and prognostic significance of EZH2 has been extensively investigated and in breast cancer it is now generally accepted to be associated with a worse prognosis [10]. Association between EZH2 expression and poor prognosis has also been reported in ductal carcinoma *in situ* (DCIS) [11]. More recently, in an analysis of the epithelial-stromal co-expression networks in breast cancer, our group has also suggested that stromal expression of EZH2 is strongly associated with breast cancer expression signatures and EZH2 expression in the epithelium of ER-negative invasive breast cancer (IBC) [12]. Also EZH2 has recently been found more frequently in the stroma of malignant phyllodes tumors (PT) when compared to normal breast tissue and borderline PT [13]. However, studies addressing the clinical relevance of EZH2 expression in benign breast disease and normal breast tissue as a biomarker of breast cancer risk have been limited by small sample sizes [14, 15]. Therefore, we performed an immunohistochemistry-based evaluation of EZH2 expression in normal breast tissue in women with biopsy-confirmed benign breast disease (BBD) in the Nurses’ Health Studies and examined the association between EZH2 expression and subsequent breast cancer risk.

## Methods

### Study subjects

This study is a nested case-control study of members of the Nurses’ Health Study (NHS) and Nurses’ Health Study II (NHS II) cohort with biopsy-confirmed BBD. The NHS is an ongoing prospective cohort study that began in 1976, when 121,700 female registered nurses between the ages of 30 and 55 years completed a mailed questionnaire. The NHS II consists of 116,609 female registered nurses who were between the ages of 25 and 42 years when the study began in 1989. In both cohorts, participants have been followed via biennial questionnaires that provide information on lifestyle factors (body mass index (BMI), reproductive history, postmenopausal hormone (PMH) use, and alcohol use) and incident disease [3, 16]. The follow-up rate for each NHS/NHS II two-year cycle has been greater than 90% of the original cohorts.

Details on the BBD diagnosis reporting on the questionnaires have been previously described [2, 6]. Briefly, the cases were women with biopsy-confirmed BBD who reported a subsequent diagnosis of breast cancer following their BBD diagnosis. Cases were diagnosed between 1976 and 1998 for the NHS and between 1989 and 1999 for the NHS II. Self-reported breast cancers were confirmed by review of medical records, and both invasive breast cancer and carcinoma *in situ* were included in the study. To reduce potential reverse causation due to subclinical tissue change, women were excluded if they had evidence of *in situ* or invasive carcinoma at biopsy or reported a diagnosis of breast cancer within 6 months of their BBD biopsy. There was a median 9 years between BBD biopsy and cancer diagnosis among the cases.

Eligible controls were women who completed the questionnaire in the same year that the breast cancer case was reported and had a previous diagnosis of biopsy-confirmed BBD, but were free from breast cancer at the time of the case (index date). Using incidence density sampling, up to four controls were selected for each breast cancer case by age at index date, year of BBD biopsy, and time since BBD biopsy. Due to considerable missing information on the laterality of the carcinoma in the cases, this information was not considered in analysis. The study was approved by the Human Subjects Research Committee of Brigham and Women’s Hospital, Boston, Massachusetts. Completion of the self-administered questionnaire was presumed to imply informed consent.

### Benign breast biopsy specimens and immunohistochemical evaluation

Eligible cases and controls were contacted for permission to obtain their BBD pathology records and biopsy specimens, and specimens were then obtained from hospital pathology departments when possible (as detailed in [2, 17, 18]). The ability to obtain biopsy blocks did not significantly differ by case and control status. Biopsy slides were independently reviewed by one of three breast pathologists. The details of this nested case-control study and the BBD assessment have been described previously [2, 6, 17]. There were 463 cases and 1853 controls, in whom the original slides had been reviewed and were eligible for the block collection. Of these, we successfully obtained BBD blocks from 177 cases and 719 controls. There were 388 participants who were eligible for the tissue microarray (TMA) construction by having one or more of the following types of benign lesions: apocrine metaplasia, non-apocrine cysts, usual ductal hyperplasia, atypical ductal hyperplasia (ADH), or atypical lobular hyperplasia (ALH). The TMAs were constructed in the Dana Farber Harvard Cancer Center Tissue Microarray Core Facility, Boston, MA, USA, by obtaining 0.6-mm cores from the targeted areas, which included lesions and up to three cores for normal terminal ductal lobular units (TDLUs) in each donor block and inserting them into the recipient TMA blocks. Three cores were obtained for 96% of the targeted areas.

For each immunohistochemical (IHC) stain, a 5-μm paraffin section was cut from each TMA block and immunostained for EZH2 (EZH2, 1:300, BD Biosciences, San Diego, CA, USA) after appropriate processing. Immunostaining was performed in a single staining run on a Leica Bond automated stainer (Leica Biosystems Inc, Nuẞloch, Germany). Positive and negative controls were included in all staining runs. Details on the antigen retrieval conditions and quantification are provided in Additional file 1. Details on ERα and Ki67 immunostaining and quantification in this cohort have been described elsewhere [6, 19]. As with previous studies using this cohort, the intensity of staining was not scored, as staining intensity can be affected by both storage time and variability in processing [20]. Owing to our samples being from across the USA over a large period, it was felt that the staining intensity would not be a reliable metric to analyze.

### Image analysis and quantification

**Fig. 1:**
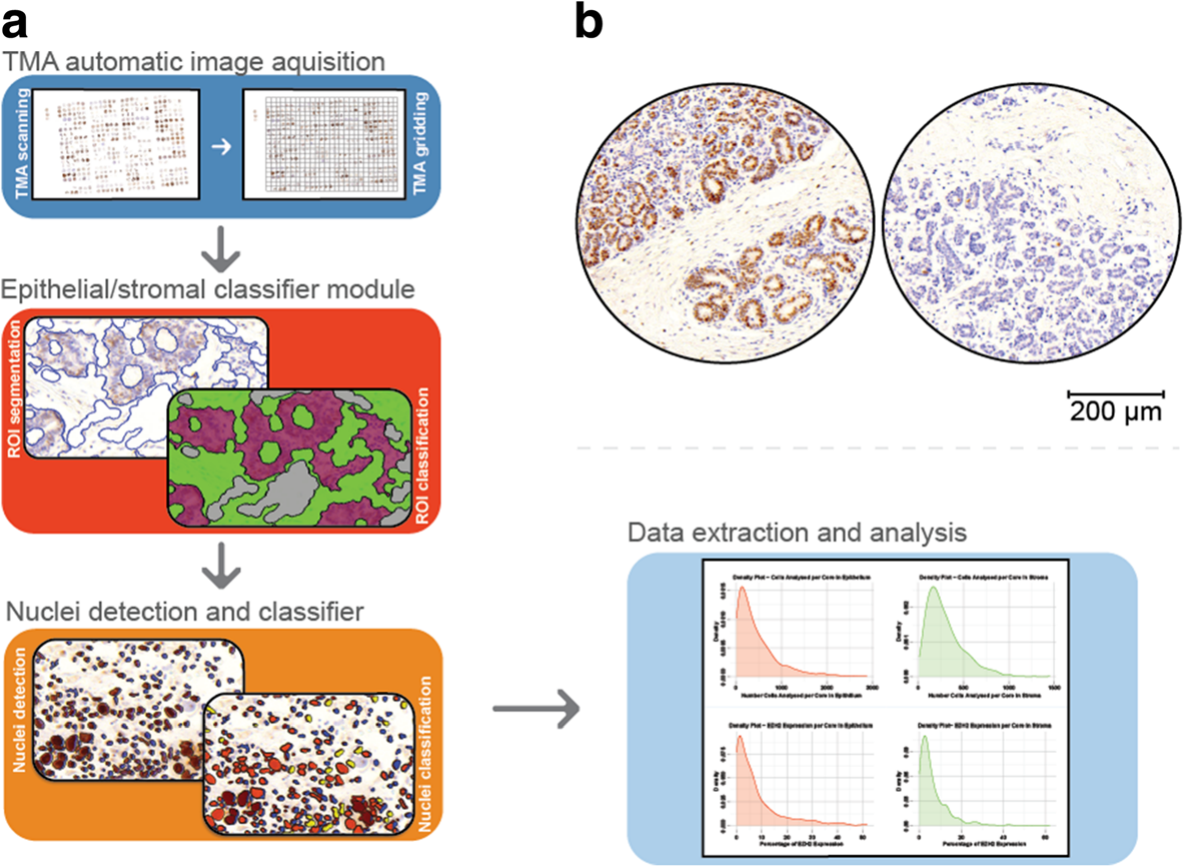
Imaging analysis of the normal breast tissue in the tissue microarrays (*TMAs*). **a** The pipeline used in this analysis consisted of the three basic stages: TMA core detection and identification matching the identification grid with the core location (*upper panel*); region of interest (ROI) segmentation, machine-learning-based training and application of the epithelium/stroma classifier (*middle panel*); and cell and nuclei detection and staining evaluation (*bottom panel*, *left side*). Using this pipeline, data were readily available for downstream analysis (*bottom panel*, *right side* - a detailed version of this panel is available in Additional file 1: Figure S2). **b** Representative images of immunohistochemical (IHC) expression of EZH2 in normal breast tissues. *Scale bar* 200 μm

Immunostaining results of each core were interpreted using an automated computational image analysis system (Definiens Tissue Studio software V4.1, Munich, Germany). EZH2 expression was recorded as the percentage of EZH2-expressing cells with nuclear expression as previously reported [14, 15]. Due to the hypothesis that EZH2 expression has a different biological significance in the stromal and epithelial compartments, the analysis pipeline included an epithelial/stromal classifier and results per compartment were also recorded (Fig. 1a). Only cells of normal TDLU’s were considered for the epithelial compartment analyses and tissue cores were excluded if containing fewer than 10 cells. The EZH2 expression in the BBD lesion was not recorded, as the capacity to target the BBD lesion in this cohort has been sub-optimal [21].

In the cell/nuclear identification and stain evaluation module, we defined an intensity and size threshold for nucleus identification and introduced an intensity threshold for nuclear stain positivity. Based on these criteria, each cell was classified as either positive or negative. The classification as a continuous scale of positive EZH2 cells was reviewed by a pathologist (FB), blinded to outcome. The image analysis results were strongly correlated with a manual 2 and 3-tier scoring system (as detailed in Additional file 1). For each woman, we estimated the mean percentage of stain-positive cells across the cores, by weighting each core by the total cell count in each of the compartments analyzed (stroma and epithelium).

### Statistical analysis

We used unconditional logistic regression analysis to describe the association between EZH2 expression and breast cancer risk. The risk estimates were presented as odds ratios (ORs) and their corresponding 95% confidence intervals (95% CIs). In the logistic regression analysis, we modeled EZH2 expression as both continuous and categorical variables (0–10%, 11–20%, >20%). Cutoff points were set based on previous reports [14, 22] and data distribution clustering based on finite normal mixture modeling (more details in Additional file 1). The logistic regression models adjusted for the study matching-factors (age at cancer diagnosis/index date, year of BBD biopsy, time since BBD biopsy), type of BBD lesion, and proliferative activity (using the Ki67 index). No imputation methods were used for handling missing information.

Potential confounding by other established breast cancer risk factors was evaluated by examining their association with EZH2 expression, though no additional covariates were included in the final models. We performed a test for trend by conducting the Wald test when including the EZH2 expression category in the model as the continuous variable. Interactions between EZH2 expression and ER and Ki-67 expression were evaluated by dichotomizing the expression about the median level among controls and performing a likelihood ratio test for the product term. Polytomous logistic regression models were utilized to evaluate EZH2 expression as a predictor of subsequent breast tumor ER expression (ER+ case vs. control, ER- case vs. control).

Statistical significance in all the analyses was assessed at the level of 0.05. The analyses were performed using SAS software (version 9.2, SAS Institute, Cary, NC, USA). All the analysis performed and reporting are compliant with the Transparent reporting of a multivariable prediction model for individual prognosis or diagnosis (TRIPOD) statement [23].

## Results

### Expression patterns of EZH2 in benign breast tissue

The percentage of EZH2-positive epithelial cells in normal TDLUs ranged from 0 to 50.00% with a median of 5.88% and an interquartile range (IQR) of 1.89–12.46%. In stroma, the percentage of EZH2-positive cells ranged from 0 to 58.39% with a median of 5.86% and an IQR of 3.09–10.91%. The intraclass correlation coefficient (ICC) across cores was 0.69 (95% CI 0.63–0.74) in the epithelium and 0.64 (95% CI 0.58–0.70) in stroma, suggesting moderately low heterogeneity in EZH2 expression across the cores. Stromal and epithelial expression of EZH2 in normal breast tissue were moderately correlated (Spearman *r* = 0.79, *p* < 0.001 (see Additional file 1: Figure S3)) and the distribution of the EZH2 expression in both the normal epithelium and stroma was similar across participants independent of the concomitant BBD diagnosis category (Additional file 1: Figure S4). Additionally, epithelial expression of EZH2 in normal breast tissue was weakly/moderately correlated with Ki67 (Spearman *r* = 0.39, *p* < 0.001) and weakly correlated with p53 (Spearman *r* = 0.21, *p* = 0.001) and AR expression (Spearman *r* = 0.18, *p* = 0.004). There was no correlation between EZH2 and ER or PR expression (Additional file 1: Table S4).

The mean age at cancer diagnosis was 53.2 years in the 74 cases included in the analysis with most developing cancer 5 to 10 years after the breast biopsy. Compared with controls, cases did not display significant differences in age-standardized characteristics (Additional file 1: Table S2). Additionally, age-standardized characteristics of controls at breast biopsy by EZH2 expression levels were also identical (Table 1).

**Table 1:** Age-standardized characteristics of the controls at breast biopsy by EZH2 expression category

	Category of EZH2 expression in normal TDLU epithelium		
	0–10% (n = 184)	11–20% (n = 55)	>20% (n = 30)
Age at cancer diagnosis/index date^a^	52.92 (8.20)	50.35 (7.20)	47.77 (8.11)
Year of BBD biopsy			
Before 1980, %	42	33	46
1980–1989, %	44	53	43
After 1989, %	14	14	11
Time from biopsy to diagnosis/index date			
0.5–4.9 years, %	48	50	45
5.0–9.9 years, %	25	23	18
10.0–14.9 years, %	19	20	34
15.0+ years, %	9	7	4
BBD category			
Non-proliferative, %	32	28	29
Proliferative without atypia, %	53	61	66
Atypical hyperplasia, %	15	12	5
Age at first birth			
Nulliparous, %	6	2	0
<25 years, %	57	42	71
25–29 years, %	30	42	13
30+ years, %	6	11	5
Missing, %	2	3	11
Duration of breastfeeding (months)^b^			
0 months, %	37	37	22
0–3 months, %	20	19	50
4–11 months, %	20	26	7
12+ months, %	21	15	18
Missing, %	2	4	2
Age at menarche (years)			
< 12, %	24	19	20
12, %	26	19	32
13, %	26	41	32
14+, %	23	21	16
Menopausal status/age at menopause (years)			
Premenopausal, %	42	43	48
< 50, %	29	30	36
50+, %	24	19	16
Missing, %	5	9	0
Postmenopausal hormone therapy use			
Never, %	26	24	33
Ever, %	30	25	16
Premenopausal, %	43	49	51
Missing, %	1	2	0
Oral contraceptive use			
Never, %	51	50	34
Ever, %	46	50	66
Missing, %	3	0	0
BMI (kg/m^2^)			
< 25.0, %	61	68	71
25.0–29.9, %	26	14	26
30.0+, %	13	17	4
Weight change since age 18 years			
Gain <2 kg, %	17	13	16
Gain 2–10 kg, %	34	39	46
Gain 10+ kg, %	39	44	32
Missing, %	10	3	5
Alcohol consumption (g/week)			
None, %	37	36	36
0.1–4.9, %	32	32	42
5.0–14.9, %	23	15	11
15.0+, %	9	17	11
Family history of breast cancer			
No family history, %	81	91	82
Family history, %	19	9	18

Values are means (SD) or percentages and are standardized to the age distribution of the study population. Values of polytomous variables may not sum to 100% due to rounding. ^a^Value is not age-adjusted. ^b^For parous women only. *EZH2* enhancer of zeste homolog 2, *TDLU* normal terminal ductal lobular units, *BMI* body mass index, *BBD* benign breast disease

### EZH2 expression patterns in normal TDLUs and subsequent breast cancer risk

Next, we examined the association between the percentage of cells positive for EZH2 in the normal TDLU epithelium and subsequent risk of breast cancer in the NHS and NHS II cohorts. When evaluating breast cancer risk by 5% increase in EZH2 expression and adjusting for matching factors between cases and controls, there was a significant increase in risk associated with EZH2 expression in epithelium (OR = 1.17, 95% CI = 1.03–1.34, *p* value = 0.021) (Table 2). Results were similar after additional adjustment for BBD subtype and proliferative activity as determined by Ki67 (OR = 1.22, 95% CI = 1.02–1.46, *p* value = 0.026). EZH2 expression in stromal cells in normal breast tissue was not significantly associated with subsequent breast cancer risk. To compare with previous studies and facilitate clinical translation of the results and estimation of EZH2 expression by a trained pathologist, we also tested the association with categories of EZH2 expression (Table 2). When using this scale, normal breast epithelium with more than 20% expression of EZH2 expression was associated with a 2.44-fold increased risk of breast cancer (OR = 2.44, 95% CI = 1.15–5.17), which was even higher when also adjusting for the BBD lesion present and for proliferative activity (OR = 2.95, 95% CI = 1.11–7.84) (Table 2).

**Table 2:** Odds ratio (95% CI) of developing breast cancer by category and 5% increase of EZH2 expression in epithelium or stroma in normal breast tissue

EZH2 expression	By category				Per 5% increase	
	≤10%	11–20%	>20%	*P* value for trend		*P* value
Epithelium						
Number of subjects (cases/controls)	48/184	10/55	16/30		74/269	
Model 1^a^	Ref.	0.70 (0.32–1.54)	2.44 (1.15–5.17)	0.078	1.17 (1.03–1.34)	0.021
Model 2^b^	Ref.	0.73 (0.3–1.63)	2.67 (1.24–5.76)	0.053	1.20 (1.04– 1.38)	0.010
Model 3^c^	Ref.	0.79 (0.32–1.96)	2.95 (1.1–7.84)	0.092	1.22 (1.02–1.46)	0.026
Stroma						
Number of subjects (cases/controls)	53/208	20/72	5/12		78/292	
Model 1^a^	Ref.	1.06 (0.58–1.96)	1.94 (0.62–6.07)	0.379	1.12 (0.93–1.35)	0.230
Model 2^b^	Ref.	1.20 (0.6 –2.23)	2.13 (0.67–6.80)	0.227	1.15 (0.95–1.39)	0.147
Model 3^c^	Ref.	1.32 (0.61–2.85)	2.45 (0.64–9.37)	0.189	1.18 (0.94–1.49)	0.157

Matching factors: age at cancer diagnosis/index date, year of biopsy and, time between benign breast disease (BBD) biopsy and cancer diagnosis/index date. ^a^Model 1: adjusting for matching factors. ^b^Model 2: adjusting for matching factors and BBD category. ^c^Model 3: adjusting for Model 2 + Ki67. *EZH2* enhancer of zeste homolog 2, *Ref.* reference

**Table 3:** Odds ratios (95% confidence interval) of developing subsequent breast cancer according to cross-classified groups of EZH2 and ER, and Ki67 in normal breast tissue

	Cases	Controls	Model 1^a^ OR (95% CI)	Model 2^b^ OR (95% CI)
Low EZH2/low ER	10	38	1.0 (Ref)	1.0 (Ref.)
Low EZH2/high ER	7	35	0.97 (0.32–2.94)	1.07 (0.35–3.31)
High EZH2/low ER	12	15	3.90 (1.27–11.99)	4.02 (1.29–12.59)
High EZH2/high ER	1	18	0.24 (0.03–2.09)	0.19 (0.02–1.70)
*P* value for interaction			0.031	0.017
Low EZH2/low Ki67	13	65	1.0 (Ref.)	1.0 (Ref.)
Low EZH2/high Ki67	7	29	1.49 (0.51–4.34)	1.56 (0.53–4.59)
High EZH2/low Ki67	12	31	2.62 (0.98–7.01)	2.40 (0.88–6.51)
High EZH2/high Ki67	22	68	1.67 (0.73–3.83)	1.78 (0.77–4.14)
*P* value for interaction			0.235	0.306

Matching factors: age at cancer diagnosis/index date, year of biopsy and, time between benign breast disease (BBD) biopsy and cancer diagnosis/index date. Enhancer of zeste homolog 2 (EZH2), estrogen receptor (ER) and Ki67 expression categorized by median expression percentage. Only EZH2 epithelial expression was considered for cross-classification. ^a^Model 1: adjusting for matching factors, ^b^Model 2: adjusting for matching factors and BBD category. *Ref.* reference

As the cases represented patients who developed both invasive and *in*-*situ* carcinoma, we next conducted sensitivity analysis including only the 62 invasive breast cancer cases and found the results to be robust when applying this restriction. In these women, >20% epithelial expression of EZH2 was associated with a 3.15-fold increase in breast cancer risk (OR = 3.15, 95% CI 1.39–7.14), relative to women with <10% expression of EZH2. There was also a 1.22-fold increased risk of breast cancer per 5% increase in epithelial EZH2 expression (OR = 1.22, 95% CI 1.06–1.41).

### Subsequent breast cancer risk and cross-classification with ER and Ki67 expression

Due to the recently reported association between proliferative activity in breast epithelium and breast cancer risk [7, 8], we next cross-classified EZH2 with Ki67 and ER expression to evaluate the potential effect of Ki67 and ER expression on the association of epithelial EZH2 expression and subsequent breast cancer risk (Table 3). For cross-classification, EZH2, ER and Ki67 expression were dichotomized at median expression. EZH2 expression displayed a significant interaction with ER expression (*p* = 0.017). Individuals with high epithelial EZH2 and lower ER expression had a fourfold increased risk of breast cancer development (OR = 4.02, 95% CI = 1.29 –12.59) compared with those who had low EZH2 and ER expression. There was no statistical association with co-expression of Ki67. However, the association between risk and EZH2 expression in cases with Ki67 below the median was close to statistical significance (OR = 2.62, 95% CI = 0.98 –7.01). The increased risk of EZH2 expression in cases with low ER expression and potentially in cases with low Ki67 expression suggests that the effect of EZH2 in breast carcinogenesis could be dependent on the biologic activity of estrogens and more pronounced in low proliferative states).

### Differential ER-positive and ER-negative breast cancer risk by EZH2 expression

EZH2 overexpression had been associated with aggressive ER-negative IBC [22, 24] and we previously identified it as one of the most connected genes in ER-negative IBC [12]. Therefore, we tested the association between EZH2 expression and the risk of a specific subtype of IBC. Despite the association between EZH2 expression and ER-negative breast cancer, we found no association between EZH2 expression and risk of ER-negative IBC. However, EZH2 expression in >20% of epithelial cells in normal breast tisse was associated with a 3.10-fold increased risk of ER-positive breast cancer (OR = 3.10, 95% CI = 1.24–7.72), when adjusting for matching factors and BBD category. Although suggesting differential biological significance of EZH2 in normal breast and in established IBC, these results should be interpreted with caution due to the small sample size in this particular analysis.

## Discussion

This is the first study, to our knowledge, to directly examine the independent association between EZH2 expression in the normal breast and risk of breast cancer, in a large prospective study. We found that EZH2 expression in normal breast epithelium was associated with breast cancer risk, particularly when >20% of the epithelial cells expressed EZH2. Importantly, this association was independent of the two most important tissue-specific risk factors in this population, the concomitant BBD lesion present and the proliferative activity as determined by Ki67 expression.

On its own, EZH2 expression in >20% of the normal breast epithelial cells was associated with a threefold increased risk of breast cancer, and a fourfold increased risk if there is concomitant low ER expression. When compared to other traditional risk factors, the magnitude of the increased risk associated with higher EZH2 expression is in between the increased risk associated with atypical hyperplasia and proliferative disease without atypia, and is well above that associated with a positive family history [5, 25]. Thus, we consider the level of risk associated with high expression of EZH2 clinically significant, especially if we consider the lack of biomarkers of risk available for this group of patients.

Two prior studies have analyzed EZH2 expression in normal breast and BBD lesions with the goal of establishing EZH2 as a potential marker of breast cancer risk [14, 15]. Ding et al., reported similar prevalence of EZH2 expression according to the pathological diagnosis and outcome (tumor development) to ours, and Kunju et al., reported an impressive area under the receiver operator curve (AUC) of 0.88 for the ability of EZH2 to predict breast cancer. However, both studies presented important limitations for translation to the clinical setting with regard to design, small total sample size (12 and 59 subjects, respectively) and a shorter follow-up time between the biopsy and the development of cancer (6.7 years) in the case group of the larger study [15].

In our secondary analysis, we found that the expression of ER appeared to modulate the effect of EZH2 on breast cancer risk. When performing cross-classification of samples using EZH2 and ER expression, women with ER expression below the median in normal epithelium and concomitant EZH2 expression above the median displayed even higher risk of breast cancer than if only using EZH2 for classification. A similar pattern of effect modification of a risk biomarker and ER expression has also been recently reported. While Ki67 expression in normal breast epithelium was associated with breast cancer risk, when cross-classification with ER was performed, high Ki67 expression was no longer predictive of risk when high ER expression was also detected [7].

Despite the similar pattern of association between risk of breast cancer and effect modification with ER, in our study there was only weak correlation between Ki67 and EZH2, and no significant change in the magnitude of the association between EZH2 and breast cancer risk when including Ki67 expression as a covariate in the model. While we cannot totally exclude the possibility that the ability of EZH2 expression to predict breast cancer is linked to cell proliferation, we favor the previously suggested hypothesis that its role as a predictor of breast cancer may depend on its effect on stem cell survival and alteration of DNA repair pathways [24, 26–30].

We acknowledge a number of limitations in this study. First, as we used TMAs, our expression data may not be representative of the whole biopsy slide or breast, due to sampling variability. Despite a very good intraclass correlation coefficient for detection of EZH2 across biopsy cores, and thus low heterogeneity in this sample, we tried to reduce this potential problem of variability by including all patients with at least three TMA cores. Given the limited sample size, we had limited power to estimate the effect sizes, thus some of the results should be interpreted cautiously, in particular the analyses using cross-classification and the differential ER status of the different tumors. As in previous studies using this cohort, women were excluded if they did not provide biopsy specimens, did not have normal TDLUs, or if there were insufficient numbers of cells in the biopsy tissue blocks. However, our ability to obtain biopsy blocks did not significantly differ by breast cancer case and control status or by established breast cancer risk factors.

Finally, the generalizability of the result could be limited to the population with concomitant BBD diagnosis in a breast biopsy, as normal breast tissue adjacent to a BBD may be different from that in women without BBD [31]. However, despite the limitations the prospective nature of this cohort with significant follow-up time and the reproducible methods used for biomarker quantification, make this study uniquely suited to examine the association between EZH2 expression in normal breast epithelium and subsequent risk of breast cancer.

## Conclusions

In conclusion, the currently available evidence suggests EZH2 expression in the normal breast can be used as a biomarker of breast cancer risk. In conjunction with previously reported breast cancer risk biomarkers by our group of investigators, such as IGF1R and Ki67, these results reinforce a new paradigm of predicting breast cancer risk by incorporating molecular markers [6, 7]. Incorporation of these markers could help stratify women into different risk groups and be the basis of tailored screening strategies, and therefore have a major impact in the care of the millions of women who undergo breast biopsies each year.

## Additional file

Additional file 1: Supplementary methods and information regarding: 1) immunostaining, antigen retrival details and quantification, including additional table with antibodies used for IHC; 2) study popoulation characteristics by case/control status at initial biopsy; 3) automatic scoring validation; 4) further details on stromal and epithelial EZH2 expression. 5) statistical analysis supplement. This file also includes 4 additional figures and 4 additional tables. (DOCX 8691 kb)

## Abbreviations

ADH: Atypical ductal hyperplasia
ALH: Atypical lobular hyperplasia
AR: Androgen expression
AUC: Area under receiver operator curve
BBD: Benign breast disease
BMI: Body mass index
CI: Confidence interval
DCIS: Ductal carcinoma in situ
ER: Estrogen receptor
EZH2: Enhancer of zeste homolog 2
IBC: Invasive breast cancer
ICC: Intraclass correlation
IGF1R: Insulin-like growth factor 1 receptor
IHC: Immunohistochemistry
IQR: Interquartile range
NHS: Nurses’ Health Studies
OR: Odds ratio
PRC2: Polycomb-repressive complex 2
PT: Phyllodes tumor
TDLUs: Normal terminal ductal lobular units
TMA: Tissue microarray

## References

[1] Chen WY, Colditz GA (2007). Risk factors and hormone-receptor status: epidemiology, risk-prediction models and treatment implications for breast cancer. Nat Clin Pract Oncol.

[2] Collins LC, Baer HJ, Tamimi RM, Connolly JL, Colditz GA, Schnitt SJ (2006). The influence of family history on breast cancer risk in women with biopsy-confirmed benign breast disease: results from the Nurses’ Health Study. Cancer.

[3] Tamimi RM, Byrne C, Colditz G a, Hankinson SE (2007). Endogenous hormone levels, mammographic density, and subsequent risk of breast cancer in postmenopausal women. J Natl Cancer Inst.

[4] Degnim AC, Visscher DW, Berman HK, Frost MH, Sellers TA, Vierkant RA, Maloney SD, Pankratz VS, de Groen PC, Lingle WL, Ghosh K, Penheiter L, Tlsty T, Melton LJ, Reynolds CA, Hartmann LC (2007). Stratification of breast cancer risk in women with atypia: a Mayo cohort study. J Clin Oncol.

[5] Hartmann LC, Sellers TA, Frost MH, Lingle WL, Degnim AC, Ghosh K, Vierkant RA, Maloney SD, Pankratz VS, Hillman DW, Suman VJ, Johnson J, Blake C, Tlsty T, Vachon CM, Melton LJ, Visscher DW (2005). Benign breast disease and the risk of breast cancer. N Engl J Med.

[6] Tamimi RM, Colditz GA, Wang Y, Collins LC, Hu R, Rosner B, Irie HY, Connolly JL, Schnitt SJ (2011). Expression of IGF1R in normal breast tissue and subsequent risk of breast cancer. Breast Cancer Res Treat.

[7] Huh SJ, Oh H, Peterson MA, Almendro V, Hu R, Bowden M, Lis RL, Cotter MB, Loda M, Barry WT, Polyak K, Tamimi RM (2016). The proliferative activity of mammary epithelial cells in normal tissue predicts breast cancer risk in premenopausal women. Cancer Res.

[8] Nassar A, Hoskin TL, Stallings-Mann ML, Degnim AC, Radisky DC, Frost MH, Vierkant RA, Hartmann LC, Visscher DW (2015). Ki-67 expression in sclerosing adenosis and adjacent normal breast terminal ductal lobular units: a nested case-control study from the Mayo Benign Breast Disease Cohort. Breast Cancer Res Treat.

[9] Wassef M, Rodilla V, Teissandier A, Zeitouni B, Gruel N, Sadacca B, Irondelle M, Charruel M, Ducos B, Michaud A, Caron M, Marangoni E, Chavrier P, Le Tourneau C, Kamal M, Pasmant E, Vidaud M, Servant N, Reyal F, Meseure D, Vincent-Salomon A, Fre S, Margueron R (2015). Impaired PRC2 activity promotes transcriptional instability and favors breast tumorigenesis. Genes Dev.

[10] Jiang T, Wang Y, Zhou F, Gao G, Ren S, Zhou C (2016). Prognostic value of high EZH2 expression in patients with different types of cancer: a systematic review with meta-analysis. Oncotarget.

[11] Knudsen ES, Dervishaj O, Kleer CG, Pajak T, Schwartz GF, Witkiewicz AK (2013). EZH2 and ALDH1 expression in ductal carcinoma in situ: complex association with recurrence and progression to invasive breast cancer. Cell Cycle.

[12] Oh E-Y, Christensen SM, Ghanta S, Jeong JC, Bucur O, Glass B, Montaser-Kouhsari L, Knoblauch NW, Bertos N, Saleh SM, Haibe-Kains B, Park M, Beck AH (2015). Extensive rewiring of epithelial-stromal co-expression networks in breast cancer. Genome Biol.

[13] Zhang Y, Liss AL, Chung E, Pierce LJ, Kleer CG (2016). Stromal cells in phyllodes tumors of the breast are enriched for EZH2 and stem cell marker expression. Breast Cancer Res Treat.

[14] Ding L, Erdmann C, Chinnaiyan AM, Merajver SD, Kleer CG (2006). Identification of EZH2 as a molecular marker for a precancerous state in morphologically normal breast tissues. Cancer Res.

[15] Kunju LP, Cookingham C, Toy KA, Chen W, Sabel MS, Kleer CG (2011). EZH2 and ALDH-1 mark breast epithelium at risk for breast cancer development. Mod Pathol.

[16] Colditz G a, Hankinson SE (2005). The Nurses’ Health Study: lifestyle and health among women. Nat Rev Cancer.

[17] Collins LC, Baer HJ, Tamimi RM, Connolly JL, Colditz GA, Schnitt SJ (2007). Magnitude and laterality of breast cancer risk according to histologic type of atypical hyperplasia. Cancer.

[18] Aroner SA, Collins LC, Connolly JL, Colditz G a, Schnitt SJ, Rosner B a, Hankinson SE, Tamimi RM (2013). Radial scars and subsequent breast cancer risk: results from the Nurses’ Health Studies. Breast Cancer Res Treat.

[19] Oh H, Eliassen AH, Wang M, Smith-Warner SA, Beck AH, Schnitt SJ, Collins LC, Connolly JL, Montaser-Kouhsari L, Polyak K, Tamimi RM (2016). Expression of estrogen receptor, progesterone receptor, and Ki67 in normal breast tissue in relation to subsequent risk of breast cancer. NPJ Breast Cancer.

[20] Atkins D, Reiffen K-A, Tegtmeier CL, Winther H, Bonato MS, Störkel S (2004). Immunohistochemical detection of EGFR in paraffin-embedded tumor tissues: variation in staining intensity due to choice of fixative and storage time of tissue sections. J Histochem Cytochem.

[21] Collins LC, Wang Y, Connolly JL, Baer HJ, Hu R, Schnitt SJ, Colditz GA, Tamimi RM (2009). Potential role of tissue microarrays for the study of biomarker expression in benign breast disease and normal breast tissue. Appl Immunohistochem Mol Morphol.

[22] Kleer CG, Cao Q, Varambally S, Shen R, Ota I, Tomlins SA, Ghosh D, Sewalt RGAB, Otte AP, Hayes DF, Sabel MS, Livant D, Weiss SJ, Rubin MA, Chinnaiyan AM (2003). EZH2 is a marker of aggressive breast cancer and promotes neoplastic transformation of breast epithelial cells. Proc Natl Acad Sci U S A.

[23] Collins GS, Reitsma JB, Altman DG, Moons KGM (2015). Transparent reporting of a multivariable prediction model for individual prognosis or diagnosis (TRIPOD): the TRIPOD statement. J Clin Epidemiol.

[24] Gonzalez ME, Li X, Toy K, DuPrie M, Ventura AC, Banerjee M, Ljungman M, Merajver SD, Kleer CG (2009). Downregulation of EZH2 decreases growth of estrogen receptor-negative invasive breast carcinoma and requires BRCA1. Oncogene.

[25] Kabat GC, Jones JG, Olson N, Negassa A, Duggan C, Ginsberg M, Kandel RA, Glass AG, Rohan TE (2010). A multi-center prospective cohort study of benign breast disease and risk of subsequent breast cancer. Cancer Causes Control.

[26] Ezhkova E, Pasolli HA, Parker JS, Stokes N, Su I, Hannon G, Tarakhovsky A, Fuchs E (2009). Ezh2 orchestrates gene expression for the stepwise differentiation of tissue-specific stem cells. Cell.

[27] Cao Q, Yu J, Dhanasekaran SM, Kim JH, Mani R-S, Tomlins SA, Mehra R, Laxman B, Cao X, Yu J, Kleer CG, Varambally S, Chinnaiyan AM (2008). Repression of E-cadherin by the polycomb group protein EZH2 in cancer. Oncogene.

[28] Gonzalez ME, Moore HM, Li X, Toy KA, Huang W, Sabel MS, Kidwell KM, Kleer CG (2014). EZH2 expands breast stem cells through activation of NOTCH1 signaling. Proc Natl Acad Sci U S A.

[29] Mondal T, Subhash S, Vaid R, Enroth S, Uday S, Reinius B, Mitra S, Mohammed A, James AR, Hoberg E, Moustakas A, Gyllensten U, Jones SJM, Gustafsson CM, Sims AH, Westerlund F, Gorab E, Kanduri C (2015). MEG3 long noncoding RNA regulates the TGF-β pathway genes through formation of RNA-DNA triplex structures. Nat Commun.

[30] Wu J, Crowe D (2015). The histone methyltransferase EZH2 promotes mammary stem and luminal progenitor cell expansion, metastasis and inhibits estrogen receptor-positive cellular differentiation in a model of basal breast cancer. Rep Oncol.

[31] Degnim AC, Visscher DW, Hoskin TL, Frost MH, Vierkant RA, Vachon CM, Shane Pankratz V, Radisky DC, Hartmann LC (2012). Histologic findings in normal breast tissues: comparison to reduction mammaplasty and benign breast disease tissues. Breast Cancer Res Treat.

