# Supplementary material for: EZH2 protein expression in normal breast epithelium and risk of breast cancer: results from the Nurses’ Health Studies
Source: Breast Cancer Res. 2017 Mar 2;19:21. doi: 10.1186/s13058-017-0817-6 (PMC5335498; doi:10.1186/s13058-017-0817-6)
Supplement: Additional file 1: — Supplementary methods and information regarding: 1) immunostaining, antigen retrival details and quantification, including additional table with antibodies used for IHC; 2) study popoulation characteristics by case/control status at initial biopsy; 3) automatic scoring validation; 4) further details on stromal and epithelial EZH2 expression. 5) statistical analysis supplement. This file also includes 4 additional figures and 4 additional tables. (DOCX 8691 kb) [file 13058_2017_817_MOESM1_ESM.docx]

**Additional file**

1. **Immunostaining, Antigen Retrieval Details and Quantification.**

Immunostaining was performed on tissue sections after deparaffinization in two 5-min changes of xylene and rehydration through graded alcohols to distilled water. After blocking endogenous peroxidase activity, sections were subjected to heat-induced epitope retrieval by heating in a vegetable steamer in Citrate buffer, pH 6.0 30min at 98ºC or EDTA (pH 8.0) for 20 min (Details in Table S1). After heat-induced epitope retrieval, the primary monoclonal antibodies were applied to the sections for 30 min or 1 hour at room temperature. The slides were then incubated with HRP-Labeled Polymer or the avidin biotin complex system (Dako Corporation, Carpinteria, CA). The reactions were completed with the Envision detection system (Dako) using 3-30 diaminobenzidine as the chromogen. For the purpose of this analysis, the quantification of AR, PR and p53 was performed using Definiens Tissue Studio software V4.1 (Munich, Germany) and recorded as a continuous scale.

| **Table S1 - Antibodies used for IHC** | | | | | | | |
| --- | --- | --- | --- | --- | --- | --- | --- |
| **Antigen** | **Source** | **Clone** | **Ref. #** | **Dilution** | **Incubation** | **Antigen retrieval** | **Detection reagent** |
| **ERα** | Thermo | SP1 | RM-9101 | 1:100 | 1 hr RT | Citrate buffer, pH 6.0 30min at 98ºC | HRP polymer |
| **PR** | Dako | PgR 636 | M3569 | 1:40 | 1 hr RT | Citrate buffer, pH 6.0 30min at 98ºC | HRP polymer |
| **AR** | Dako | AR441 | M3562 | 1:200 | 30 min RT | EDTA, pH 9.0 20min at 98ºC | HRP polymer |
| **p53** | ImmunoTech | 1767 | #1767 | 1:250 | 1 hr RT | Citrate buffer, pH 6.0 30min at 98ºC | HRP polymer |
| **Ki-67** | Vector | SP6 | VP-RM04 | 1:250 | 30 min RT | EDTA, pH 9.0 20min at 98ºC | HRP polymer |
| **EZH2** | BD Biosciences | 11/EZH2 | 612667 | 1:300 | 1 hr RT | Citrate buffer, pH 6.0 30min at 98ºC | SABC |

1. **Study Population characteristics by case/control status at initial biopsy**

| **Table S2 - Age-standardized characteristics of the study population at benign breast biopsy** | | |
| --- | --- | --- |
|  | **Controls (n=269)** | **Case (n=74)** |
| Age at cancer diagnosis/index date* | 51.82(8.16) | 53.18(9.05) |
| Year of BBD biopsy |  |  |
| Before 1980, % | 40 | 42 |
| 1980-1989, % | 47 | 48 |
| After 1989, % | 13 | 10 |
| Time from biopsy to dx/index date |  |  |
| 0.5-4.9 years, % | 48 | 24 |
| 5.0-9.9 years, % | 24 | 37 |
| 10.0-14.9 years, % | 20 | 21 |
| 15.0+ years, % | 8 | 18 |
| BBD category |  |  |
| Non-proliferative, % | 30 | 24 |
| Proliferative without atypia, % | 56 | 52 |
| Atypical hyperplasia, % | 14 | 24 |
| Age at first birth |  |  |
| Nulliparous, % | 4 | 3 |
| <25 years, % | 55 | 40 |
| 25-29 years, % | 31 | 45 |
| 30+ years, % | 7 | 8 |
| Missing, % | 4 | 4 |
| Duration of breastfeeding (mo.) |  |  |
| 0 months, % | 36 | 33 |
| 0-3 months, % | 22 | 19 |
| 4-11 months, % | 20 | 29 |
| 12+ months, % | 19 | 18 |
| Missing, % | 2 | 2 |
| Age at menarche (yr.) |  |  |
| -12, % | 23 | 29 |
| 12, % | 25 | 30 |
| 13, % | 30 | 27 |
| 14+, % | 22 | 14 |
| Age at menopause (yr.) |  |  |
| Premenopausal, % | 42 | 51 |
| <50, % | 30 | 24 |
| 50+, % | 23 | 20 |
| Missing, % | 5 | 5 |
| Postmenopausal hormone therapy use |  |  |
| Never, % | 27 | 23 |
| Ever, % | 28 | 27 |
| Premenopausal, % | 44 | 50 |
| Missing, % | 1 | 0 |
| Oral contraceptive use |  |  |
| Never, % | 49 | 40 |
| Ever, % | 48 | 58 |
| Missing, % | 2 | 2 |
| BMI (kg/m2) |  |  |
| <25.0, % | 64 | 68 |
| 25.0-29.9, % | 23 | 23 |
| 30.0+, % | 13 | 10 |
| Weight change since age 18 |  |  |
| Gain <2kg, % | 17 | 22 |
| Gain 2-10kg, % | 35 | 34 |
| Gain 10+kg, % | 39 | 35 |
| Missing, % | 8 | 9 |
| Alcohol consumption (g/week) |  |  |
| None, % | 36 | 40 |
| 0.1-4.9, % | 34 | 34 |
| 5.0-14.9, % | 20 | 26 |
| 15.0+, % | 11 | 0 |
| Family history of breast cancer |  |  |
| No Family History, % | 84 | 72 |
| Family History, % | 16 | 28 |
| *Value not age adjusted | | |

1. **Automatic Score Validation**

To assess the correlation between automated and manual scoring, a single IHC stained-TMA slide was randomly chosen and the cores manually scored. Continuous data was transformed in a two and three-tier classification using univariate clustering based on finite normal mixture modeling using the complete dataset (Mclust 5.1 package, R 3.2.2). This automatic, data-driven classification method resulted in similar categories for epithelial and stromal expression, briefly: 0, >10% expression and 1, 10% expression for the two-tier classification and 0, >10% expression, 1, 10-20% expression and 2, ≤20% expression for the three-tier classification scoring. Afterwards, a trained pathologist (FB) manually scored one TMA slide (containing 269 cores) using the categories described above for the epithelial and stromal compartments (Supplementary materials figure 1). All scoring was done blindly to the outcome or patient data. The correlation between scoring methodologies was high/moderate with Spearman’s correlation rho values as it follows: for epithelium 0.79 and 0.73 (for 3 and 2 tier classification, respectively and, for stroma 0.58 (for both the 3 and 2 tier classification). Additionally, we evaluated the accuracy of the 2 tier manual classification for epithelial cells and stromal cells classification which was 84.36% and 80.00%, respectively (table S3).


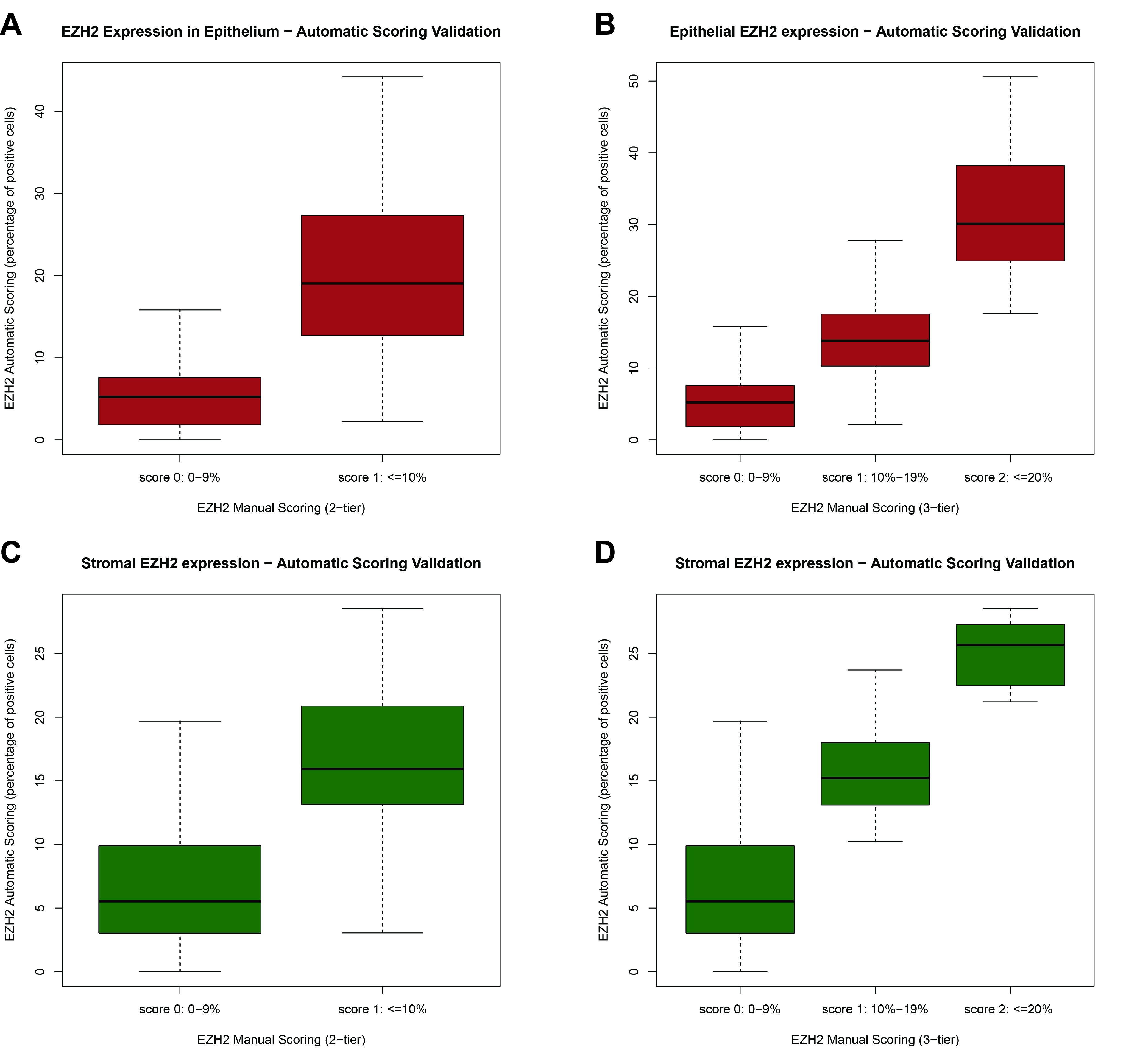


**Figure S1. Boxplots of the percentage of cells expressing EZH2 according to the manual classification category.** The percentage of positive cells as obtained by the automatic quantification system is depicted in the y-axis with the manual classification category on the x-axis for epithelial cells with a 2 and 3 tier manual classification (A and B, respectively) and, for stromal cells with a 2 and 3 tier manual classification (C and D, respectively).

| **Table S3 - Automatic Score Validation Summary** | | | |
| --- | --- | --- | --- |
| **Scoring** | **Sensitivity** | **Specificity** | **Accuracy** |
| Epithelium: Manual Vs Automatic | 0.87 | 0.82 | 0.84 |
| Stroma: Manual Vs Automatic | 0.96 | 0.76 | 0.80 |

1. **Stroma and Epithelial EZH2 Expression**

**
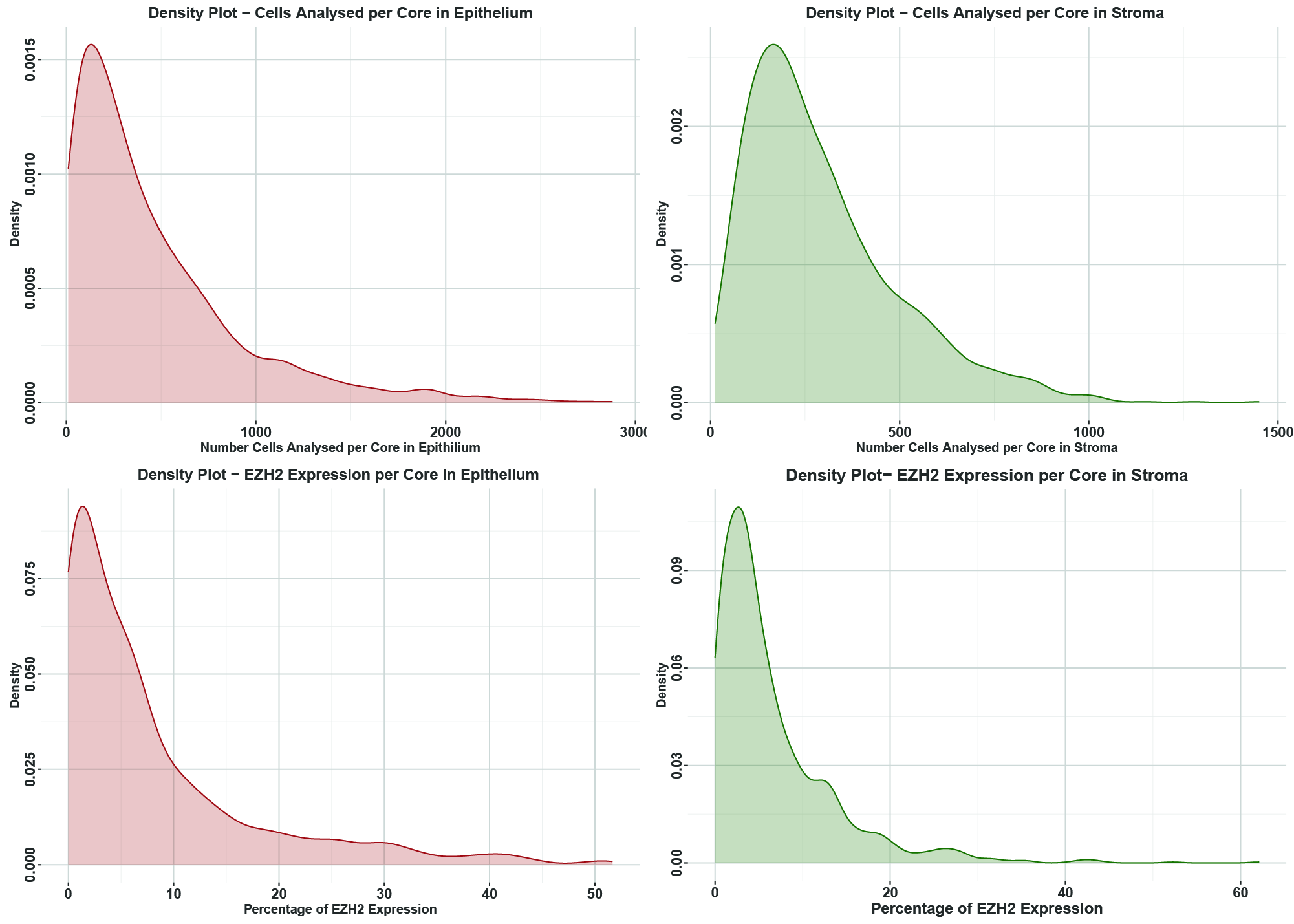
**

**Figure S2. EZH2 expression and number of cells analyzed in epithelium and stromal areas.** Density plots showing the number of cells analyzed per area type (left column) and EZH expression (right column).

**
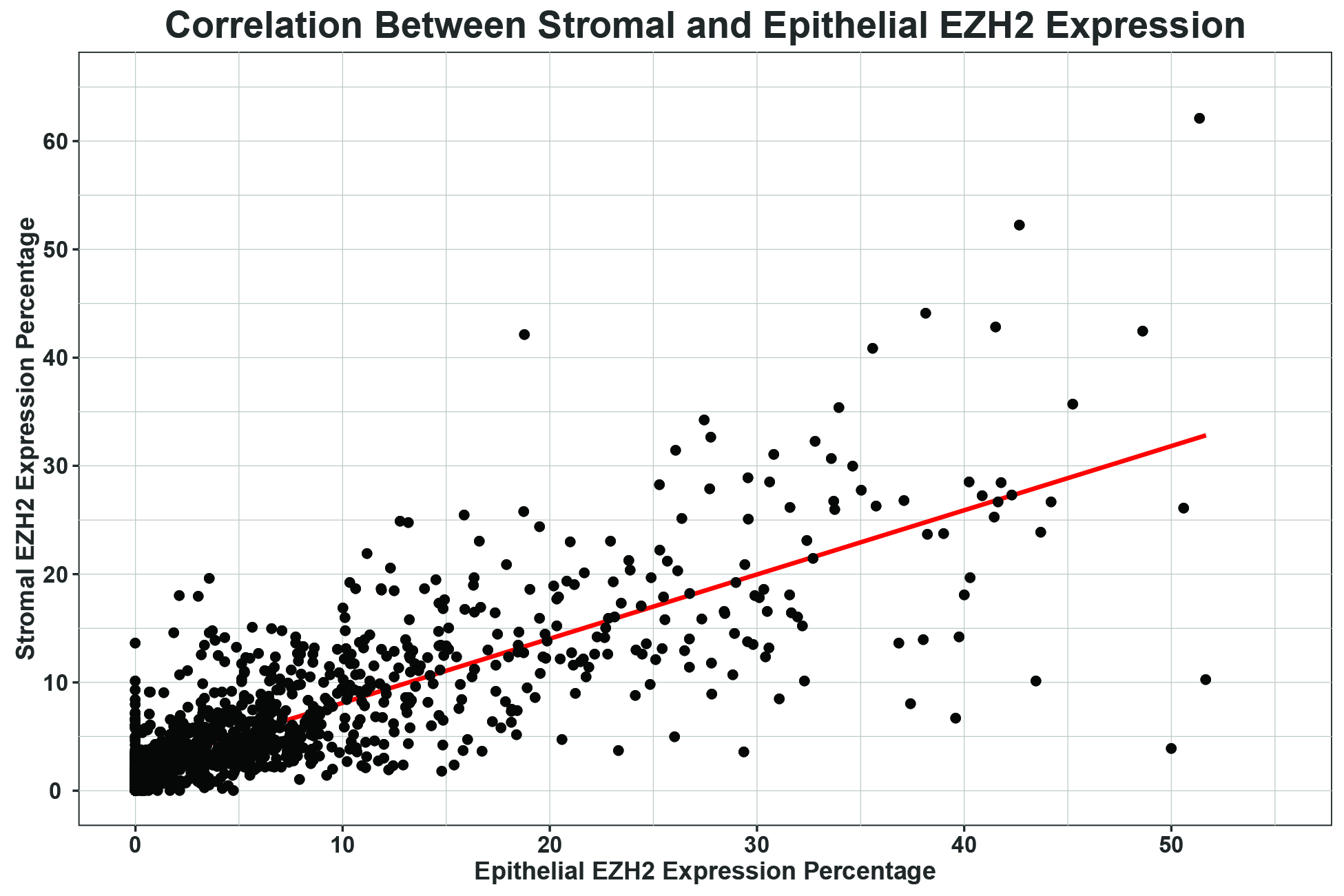
**

**Figure S3. Scatter plot with the correlation between EZH2 expression in epithelial and stroma cells.** The Pearson’s correlation between the percentage of positive cells expressing EZH2 in the epithelium and stroma was 0.79 (p-value <0.001) in the1270 cores included in the analysis.

| **Table S4 - Correlations with EZH2 expression in normal breast tissue** | | | | | | | |
| --- | --- | --- | --- | --- | --- | --- | --- |
|  | **Epithelium EZH2 expression** | | |  | **Stromal EZH2 expression** | | |
| **Marker** | **Individuals (n)** | **Spearman’s r** | **p-value*** |  | **Individuals (n)** | **Spearman’s r** | **p-value*** |
| ER | 168 | -0.14 | 0.073 |  | 178 | -0.17 | 0.023 |
| Ki67 | 302 | 0.39 | <0.001 |  | 319 | 0.32 | <0.001 |
| PR | 190 | 0.10 | 0.170 |  | 203 | 0.04 | 0.602 |
| P53 | 253 | 0.21 | 0.001 |  | 270 | 0.24 | <0.001 |
| AR | 241 | 0.18 | 0.004 |  | 255 | 0.19 | 0.003 |
| * Reported p-value not adjusted. If using Bonferroni correction, p-value should be considered significant at 0.01 level | | | | | | | |

**
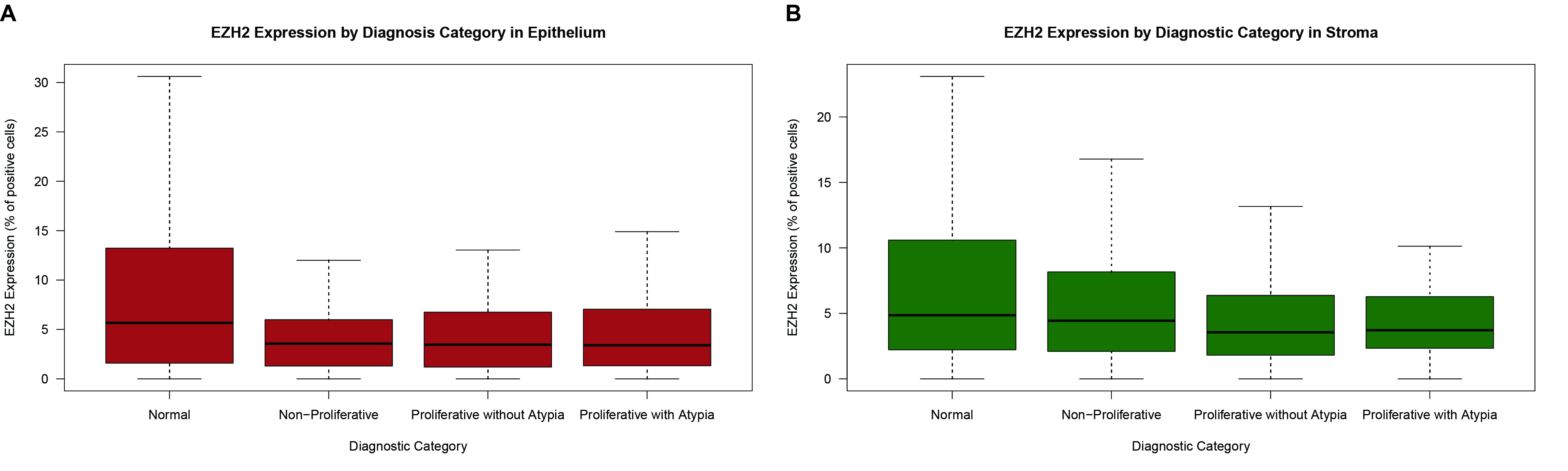
**

**Figure S4. Boxplots of the percentage of cells expressing EZH2 in normal breast tissue according to concurrent diagnosis.** The expression levels of EZH2 in both the epithelium (A) and stroma (B) were comparable independently of the concurrent diagnosis in the breast biopsy in this study (p-value=n.s.).

1. **Statistical Analysis Supplement**

We adjusted for the following matching factors age at breast cancer diagnosis or index date (<45, 45-54, 55+ years), year of BBD biopsy (before 1980, 1980-1989, after 1989), and time between BBD biopsy and cancer diagnosis or index date (0.5-4.9, 5.0-9.9, 10.0-14.9, 15.0+ years). We considered the following potential confounders measured at the time of BBD biopsy: BBD type (non-proliferative, proliferative without atypia, atypical hyperplasia), age at first birth (nulliparous, <25, 25-29, 30+years, missing, duration of breastfeeding (0, 0-3, 4-11, 12+ months, missing), age at menarche (<12, 12–13, or >13 years), age at menopause (premenopausal, <50, 50+ years, missing), menopausal hormone therapy (never used hormones, ever used hormones, missing), oral contraceptive use (never used, ever used), body mass index (<25.0, 25.0-29.9, 30.0+ kg/m^2^), weight change since age 18 (gain <2kg, gain 2-10kg, gain 10+kg, missing), alcohol consumption (none, 0.1-4.9, 5.0-14.9, 15.0+ g/week) , and a family history of breast cancer (yes or no).
